# Interplay between Basic Residues of Hepatitis C Virus Glycoprotein E2 with Viral Receptors, Neutralizing Antibodies and Lipoproteins

**DOI:** 10.1371/journal.pone.0052651

**Published:** 2012-12-27

**Authors:** George Koutsoudakis, Jakub Dragun, Sofia Pérez-del-Pulgar, Mairene Coto-Llerena, Laura Mensa, Gonzalo Crespo, Patricia González, Miquel Navasa, Xavier Forns

**Affiliations:** Liver Unit, Hospital Clínic, Institut D'Investigacions Biomèdics August Pi i Sunyer, Centro de Investigación Biomédica en Red de Enfermedades Hepáticas y Digestivas, Barcelona, Spain; University of Modena & Reggio Emilia, Italy

## Abstract

Positively-charged amino acids are located at specific positions in the envelope glycoprotein E2 of the hepatitis C virus (HCV): two histidines (H) and four arginines (R) in two conserved WHY and one RGERCDLEDRDR motifs, respectively. Additionally, the E2 hypervariable region 1 (HVR1) is rich in basic amino acids. To investigate the role(s) of these residues in HCV entry, we subjected to comparative infection and sedimentation analysis cell culture-produced (HCVcc, genotype 2a) wild type virus, a panel of alanine single-site mutants and a HVR1-deletion variant. Initially, we analyzed the effects of these mutations on E2-heparan sulfate (HS) interactions. The positive milieu of the HVR1, formulated by its basic amino acids (key residues the conserved H^386^ and R^408^), and the two highly conserved basic residues H^488^ and R^648^ contributed to E2-HS interactions. Mutations in these residues did not alter the HCVcc-CD81 entry, but they modified the HCVcc-scavenger receptor class B type I (SR-BI) dependent entry and the neutralization by anti-E2 or patients IgG. Finally, separation by density gradients revealed that mutant viruses abolished partially or completely the infectivity of low density particles, which are believed to be associated with lipoproteins. This study shows that there exists a complex interplay between the basic amino acids located in HVR1 and other conserved E2 motifs with the HS, the SR-BI, and neutralizing antibodies and suggests that HCV-associated lipoproteins are implicated in these interactions.

## Introduction

Hepatitis C virus (HCV) is a small enveloped positive-strand RNA virus that belongs to the *Flaviviridae* family [1]. HCV possesses two envelope glycoproteins (gps), designated E1 and E2, which drive the viral component during the infection of the hepatic cells [2]. Initial attachment of HCV on hepatocytes occurs via binding of E2 with highly sulfated heparan sulfate (HS) [3], [4]. These unspecific interactions aid the concentration of HCV on the surface of hepatic cells for further interactions with the subsequent specific receptors. CD81 has been the first molecule identified to interact with a soluble truncated form of E2 [5]. Several amino acids critical for E2-CD81 interaction have been identified throughout the CD81 large extracellular loop and E2 by biochemical assays [6], [7]. Recently, the development of the HCV pseudoparticle (HCVpp) [8], [9] and the HCV cell-culture (HCVcc) systems [10], [11], [12] has provided valuable tools to study HCV-receptors interactions in a more natural context.

Scavenger receptor class B type I (SR-BI) was identified as a potential HCV receptor via its extracellular loop interactions with E2 [13]. Recent data, though, are controversial. SR-BI natural ligands involve a variety of lipoproteins (HDL, LDL, oxidized LDL) which can modulate HCV infection: HDL is able to enhance HCVpp and HCVcc infections [14], [15] whereas oxidized LDL acts in an inhibitory way [16]. Interestingly, by using serum-derived HCV, it has been suggested that the virus associated ApoB-containing lipoproteins rather than the E2 interact with SR-BI [17]. Finally, Grove *et al.*, identified a residue in E2 (G451) that determined SR-BI dependency and sensitivity to neutralizing antibodies [18].

HCV circulates in the blood in association with LDL and VLDL [19], thus constituting the so-called lipo-viral particles (LVPs). LVPs exhibit low densities (≤1.08 g/ml) and are highly infectious. Increasing evidence arises from studies demonstrating that HDL and likely other HCV-associated lipoproteins promote HCV entry via receptors-lipoproteins interactions and reduce the neutralizing effect of anti-HCV antibodies [14], [20], [21]. The hypervariable region 1 (HVR1) of E2 has been implicated in these mechanisms [22]

The envelope gps of HCV exhibit a high degree of genetic heterogeneity, especially in the HVR1 of E2 [23]. However, positively-charged amino acids appear to be predominant in several HVR1 positions and may contribute to virus entry [24]. Moreover, basic amino acids at conserved regions along E2 appear to play a role in E2-HS binding *in vitro*. These regions have been characterized as putative glycosaminoglycan (GAG)-binding sites and are composed of the following motifs: ^484^PYCWHYAP^491^, ^610^DYPYRLWHYPC^620^ and ^648^RGERCDLEDRDR^659^. Basic amino acids H^488^, H^617^, R^648^, R^651^, R^657^ and R^659^ are known to play a major role in this type of binding [25].

The aim of this study was to analyze the role(s) played by basic amino acids located in HVR1, as well as in the putative GAG-binding sites found in E2 with respect to their interactions with viral receptors during HCV entry. Unravelling the complex interactions between HCV envelope glycoproteins and the various cellular receptors could help us not only to fully understand the highly orchestrated events that occur during HCV entry, but also to design specific and highly potent anti-HCV therapeutic strategies.

## Materials and Methods

### Ethics statement

The Investigation and Ethics Committee of Hospital Clinic Barcelona approved our protocol including the use of human samples, and conformed to the ethical guidelines of the 1975 Declaration of Helsinki. Written informed consent was obtained from all patients included in this study.

### Cell lines

Huh-7.5 cells were grown in Dulbecco's modified eagle medium (DMEM; Invitrogen, Carlsbad, CA) supplemented with 10% fetal bovine serum, 2 mM L-glutamine, non-essential amino acids, and 100 U/ml of streptomycin/penicillin (DMEM complete). Cells were grown in 37°C 5% CO_2_ in a water-jacketed incubator.

### Antibodies

A detailed list of antibodies used in this study is given in Information S1.

### IgG isolation from patient or normal sera

Patient or normal blood (HCV+ or HCV−, respectively) was collected in a Vacutainer® Rapid Serum Tube (Becton Dickinson, Franklin Lakes, NJ) and sera were separated after centrifugation at 1,700× *g* for 10 min. 150 µl serum was used for total IgG isolation by using a protein A HP SpinTrap™ (GE Healthcare, Waukesha, WI) according to the manufacturer's instructions.

### Site-directed mutagenesis

Alanine mutants were introduced into the HC-J6CH E1E2 expression plasmid (pcDNA3.1-ΔcE1E2-J6CH) using a PCR-based GENEART® Site-Directed Mutagenesis System (Invitrogen Eugene, OR). A detailed description of this method is available in the Information S1 section.

### In vitro transcription, electroporation of HCV RNAs, generation of HCVcc stocks, luciferase assays, quantification of HCV core protein, RT-qPCR and determination of virus titers in cell culture supernatants

These methods were employed as previously described [26].

### Preparation of cell lysates, PAGE and Western blot

Huh-7.5 cells were electroporated with Jc1 WT or mutant RNAs. 72 h post electroporation, cells were washed with PBS and lysed on ice with lysis buffer (0.5% Triton X-100 in 50 mM Tris-HCl [pH 7.5], 150 mM NaCl, 5 mM EDTA) supplemented with protease inhibitor (1 mM PMSF) for 30 min. Cell lysates were clarified by centrifugation (30 min 20,000× *g*) at 4°C. Total protein in the lysates was quantified with a Lowry protein assay and equal protein amounts (30 µg) for each sample were diluted into sample buffer (160 mM Tris, pH 6.7, 2% SDS, 10% glycerol, 0.004% bromophenol blue, 50 mM DTT). Samples were heated at 70°C for 10 min and then loaded onto a NuPAGE, Novex 4–12%, Bis-Tris Midi gel (Invitrogen, Eugene, OR). Following electrophoresis, proteins were transferred onto an Immobilon-P PVDF membrane (Millipore, Billerica, MA) using semi-dry blotter unit (Sigma-Aldrich, St. Louis, MO). Membranes were blocked with 5% non-fat milk in PBS, 0.05% Tween-20 (blocking solution) for 1 h at RT. Incubation with the primary antibody anti-E2 (BDI167, 0.5 µg/ml) or anti-β-actin (0.01 µg/ml) was performed in blocking solution overnight at 4°C. Blots were washed five times for 10 min in washing solution (0.05% Tween-20 in PBS), incubated for 1 h with anti-mouse HRP-conjugated secondary antibody in blocking solution (1∶5,000, Sigma-Aldrich, St. Louis, MO), and washed as described above. Antibody-protein complexes were detected using the SuperSignal® West Femto Maximum Sensitivity Substrate Kit (Thermo Fischer Scientific Inc., MA) with a LAS4000 Image acquisition system (GE Healthcare Life Sciences, Uppsala, Sweden).

In the case of ApoE analysis in density fractions, 10 µl of each fraction were incubated with 2.5 µl sample buffer, loaded onto a NuPAGE gel as described previously, electrophorized and transferred to a PVDF membrane. Immobilized ApoE proteins were detected with the anti-ApoE (Abnova, PAB9972, 0.1 µg/ml).

### E2 conformational antibody immunoprecipitation

500 µg of each cell lysate described above were incubated with 1 µg of AR3A conformational antibody overnight at 4°C with gentle agitation. Protein G Sepharose 4 Fast Flow beads (GE Healthcare Life Sciences, Uppsala, Sweden) were rinsed with lysis buffer and 40 µl of beads was added to each AR3A-antibody pre-treated cell lysate. Sepharose beads-lysates-antibodies complexes were incubated for 3 h with gentle rocking at 4°C. Complexes were washed 3 times with lysis buffer. Finally, beads were spun at full-speed and supernatants were discarded. 25 µl SDS-PAGE loading buffer was added to the beads, and samples were boiled for 10 min at 70°C and were then analyzed by PAGE and western blotting as described above. Bands density quantification was performed by the ImageJ Software.

### HCVcc infections and entry inhibitions

HCVcc infections were employed as previously described [4] with normalized HCVcc-containing supernatants (1,000 fmol/l core). To facilitate quantification of infection, we utilized the bicistronic Jc1 luciferase reporter construct Luc-Jc1 in our infection analyses. Huh-7.5 cells were seeded in 12 well plates, 8×10^4^ cells/well, 18 h prior to inoculation with normalized HCVcc-containing supernatants. In the case of heparin (heparin sodium salt from porcine intestinal mucosa, Calbiochem, Darmstadt, Germany), chondroitin (chondroitin sulfate C sodium salt from shark cartilage, Sigma-Aldrich, St. Louis, MO), AR3A or patient IgG neutralizations, GAGs or antibodies were pre-incubated with HCVcc-containing supernatants for 1 h prior to inoculation, at RT with agitation. Anti-CD81 or anti-SR-BI antibodies were added directly to supernatants simultaneously to inoculations. Infections were maintained for 6 h at 37°C 5% CO_2_ in a water-jacketed incubator with gentle rocking. Cells were lysed 72 h post infection and luciferase activity was measured. Infections were performed always in duplicate wells measured in duplicates (n = 4). Results are given as percentage relative to control infections as follows: heparin relative to chondroitin, mouse mAbs relative to mouse IgG_1_ isotype control, human polyclonal and mAbs relative to normal human IgG, rat polyclonals relative to normal rat polyclonal IgG and goat polyclonal to goat normal serum control.

### RNA Interference Assay

Commercially available siRNA pools targeting SR-BI as well as control nontargeting siRNAs were purchased from Novus biologicals (Littleton, CO). Huh-7.5 cells in each well of a 12-well cell culture plate were transfected with 50 nM of each siRNA using INTERFERin™ (Polyplus-transfection SA, Illkrich, France) following the manufacturer's protocol. Silencing of SR-BI expression was assessed by flow cytometry 48 hours after transfection as described in Information S1. 48 hours after transfection, cells were incubated with HCVcc, and HCV infection was assessed as described previously.

### Density gradient fractionation

Density gradient centrifugation was performed as has been previously described [27]. Briefly, viruses were separated by overnight centrifugation through a 0% to 40% iodixanol step gradient at 154,000× *g* in a SW41-T1 swing-out rotor at 4°C using a Sorvall Ultra WX100 centrifuge and 12 fractions of 1 ml were collected from the top.

### Statistical analyses

The results are presented as means ± standard deviation (SD). The statistical comparison between two groups was made by an unpaired-*t* test. *p value<0.05, **p value<0.01 and ***p value<0.001 were considered to indicate a significant difference.

## Results

### Analyses of GAG-binding sites and basic residues in E2 sequences

Our experiments were performed in the context of genotype 2a (Jc1 chimera [28], HC-J6CH E2, Acc. No. AF177036) HCVcc virus. The prototype E2 sequence (H77 isolate, accession Number AF009606) contains 363 amino acids. However, HC-J6CH E2 contains 367 amino acids. To simplify our analyses, amino acid numbers refer to positions in the polyprotein sequence of the H77 prototype isolate.

**Figure 1:**
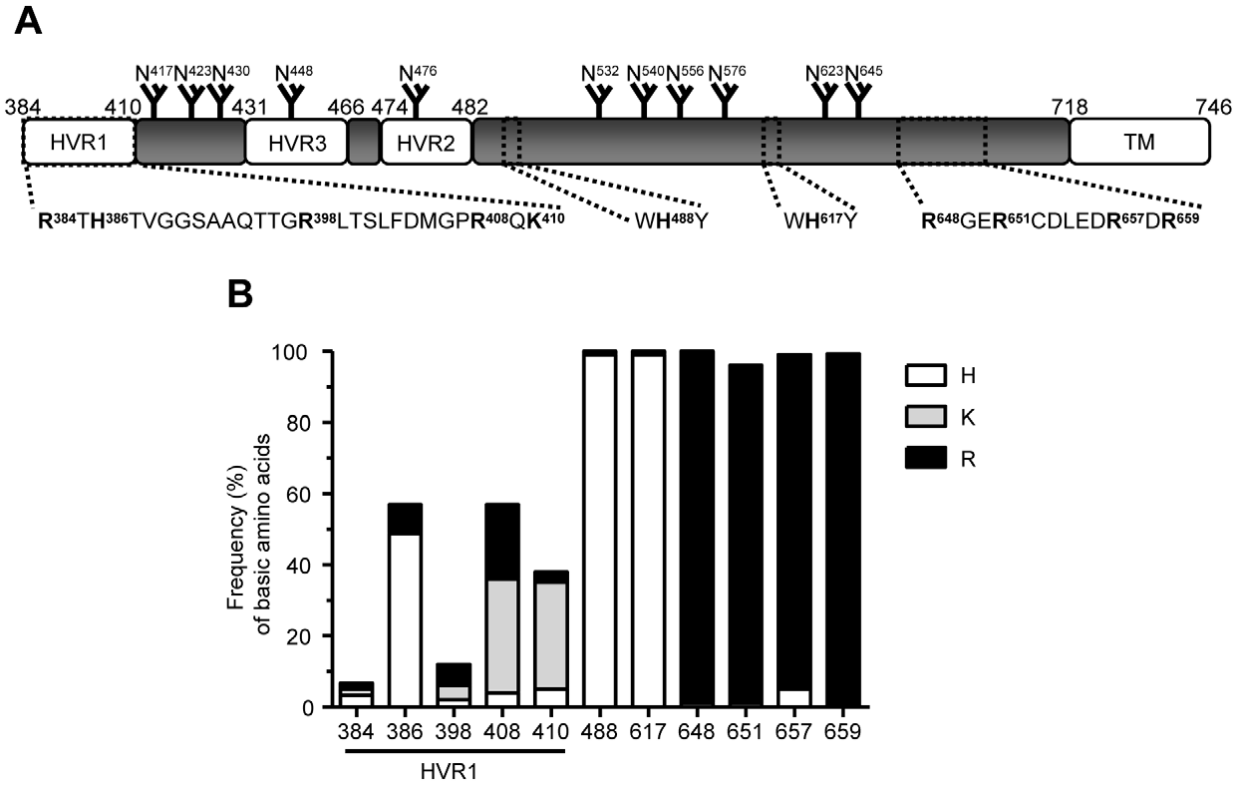
HCV E2 glycoprotein contains conserved basic residues in different regions. (A) Scheme of E2 putative GAG-binding sites and other regions important for entry and for proper protein folding. Amino acid numbers refer to positions in the polyprotein sequence of the H77 prototype isolate. N: glycosylation sites, HVR1, 2, 3: hyper-variable region 1, 2, 3, TM: transmembrane domain. (B) Frequency of basic residues at the positions analyzed in this study. The height of the box in each bar indicates the frequency of histidine (H, white box), lysine (K, light grey box) and arginine (R, dark grey box). The frequency of the basic residues at each position was calculated by dividing the number of basic residues by the total number of sequences (2073 sequences) and is expressed as a percentage.

The HC-J6CH isolate contains five basic amino acids in the HVR1: R^384^, H^386^, R^398^, R^408^ and K^410^. Additionally, it contains the H and R conserved residues in the putative GAG-binding sites mentioned in the *Introduction*. Figure 1A depicts the scheme of an E2 protein, indicating the HVR1, 2 and 3 regions, the putative GAG-binding sites and sites for N glycosylation.

To assess the conservation of those basic residues between the HC-J6CH E2 proteins and the natural E2 variants, we analyzed the alignment of full-length E2 proteins, available in the European HCV database (http://euhcvdb.ibcp.fr/euHCVdb/). The alignment contained 2073 full-length E2. We calculated the frequencies of all basic amino acids (H, K, or R) at these positions (Fig. 1B). The basic residues at positions 384 and 398 presented a low frequency (6.7 and 7.5%, respectively). Nonetheless, those at positions 386, 408, and 410 occurred at higher frequencies (57.0, 54.4 and 34.5%, respectively) with H, K, and K being the predominant amino acids found at each position, respectively. Finally, H^488^, H^617^, R^648^, R^651^, R^657^ and R^659^ were almost completely conserved.

### Generation of E2 glycoprotein mutants

A panel of point alanine site-directed mutants was generated and divided into 2 groups: Group A referred to mutants relevant to the HVR1 while group B contained mutants at the putative GAG-binding sites. To expand our study in the HVR1, we generated a mutant E2 possessing an alanine substitution in all basic amino acids of the HVR1 (“basic-” mutant), as well as a deletion mutant (ΔHVR1). Finally, by introducing an alanine at position 369 and simultaneously mutating the K^370^ into an alanine, the α-helix of the E1 transmembrane (TM) domain was destabilized, thereby affecting E1E2 heterodimerization (E1AA mutant) [29]. As this mutant causes E1E2 aggregation and degradation, it served as a negative control. Table 1 summarizes the E2 mutants.

**Table 1:** Mutations of conserved basic amino acids of HCV E2 glycoprotein.

Mutations		Isolate HC-J6CH	
	aa position	Amino acid level	Nucleotide level
Group A	384	R/A	CGC/GCC
	386	H/A	CAT/GCT
	398	R/A	CGC/GCC
	408	R/A	AGG/GCG
	410	K/A	AAA/GCA
	basic-	R/A, H/A, R/A, R/A, K/A	CGC/GCC, CAT/GCT, CGC/GCC, AGG/GCG, AAA/GCA
	ΔHVR1	Δ27 aa^a^	Δ81 nt^b^
Group B	488	H/A	CAC/GCC
	617	H/A	CAT/GCT
	648	R/A	CGT/GCT
	651	R/A	CGT/GCT
	657	R/A	AGA/GCA
	659	R/A	AGA/GCA
E1AA	369, 370	A insertion, K/A	GCG insertion, AAG/GCA

a Deletion of 27 aa from the N-terminal part of the E2 protein.

b Deletion of 81 nt from the 5′ terminus of the E2 gene.

**Figure 2:**
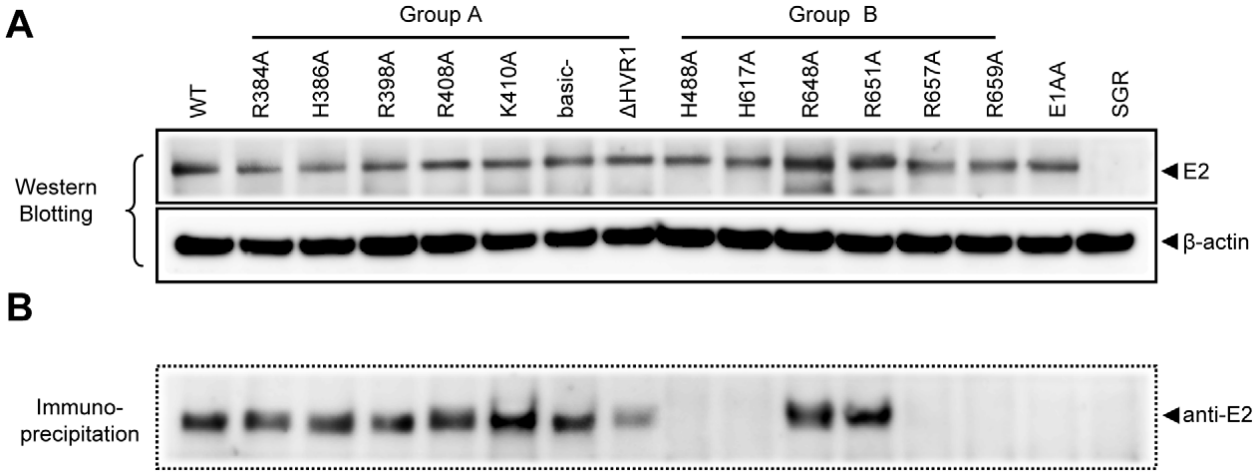
Expression and conformation analysis of HCV E2 glycoprotein in cell lysates. (A) Expression analysis of HC-J6CH E2 protein by anti-E2 Western blot (B) E2 conformation analysis by E2 immunoprecipitation, as well as subsequent E2 detection by Western blot. β-actin detection was used as a loading control, while cell lysates from cells electroporated with JFH1 subgenomic replicons (SGR) were used as a negative control in both Western blot and immunoprecipitation experiments.

Adequate expression of the various E2 mutants was examined by Western blot analysis in lysates derived post-electroporation from Huh-7.5 cells with WT or mutants. When probed with anti-E2 antibody, a band of ∼68 kDa was detected, which corresponded to the size of the HCV E2 protein (Fig. 2A). Lysates from electroporated cells with a HCV subgenomic replicon (SGR [30]), that does not contain glycoproteins, were used to control anti-E2 specificity. Overall, neither the alanine mutations nor the HVR1 deletion markedly affected E2 protein expression.

Next, we investigated whether any of these alanine substitutions had any effect on E2 conformation. E2 conformation affects proper E1E2 heterodimerization, which is essential in mediating HCV infectivity [31]. To this end, E2 present in the Huh-7.5 cell lysates was pulled down with a conformational anti-E2 antibody [32]. Immunocomplexes were analyzed by western blot for the presence of E2 using monoclonal anti-E2 antibodies (Fig. 2B). Group A mutants (except ΔHVR1) did not markedly affect E2 conformation, according to the amount of E2 immunoprecipitated by this method. For the group B mutants, only mutants R648A and R651A permitted sufficient E2 detection by immunoprecipitation while the H488A, H617A, R657A and R549A mutations almost completely abrogated the E2 conformation.

### Effect of E2 alanine substitutions on infectivity and heparin neutralization of HCVcc

**Figure 3:**
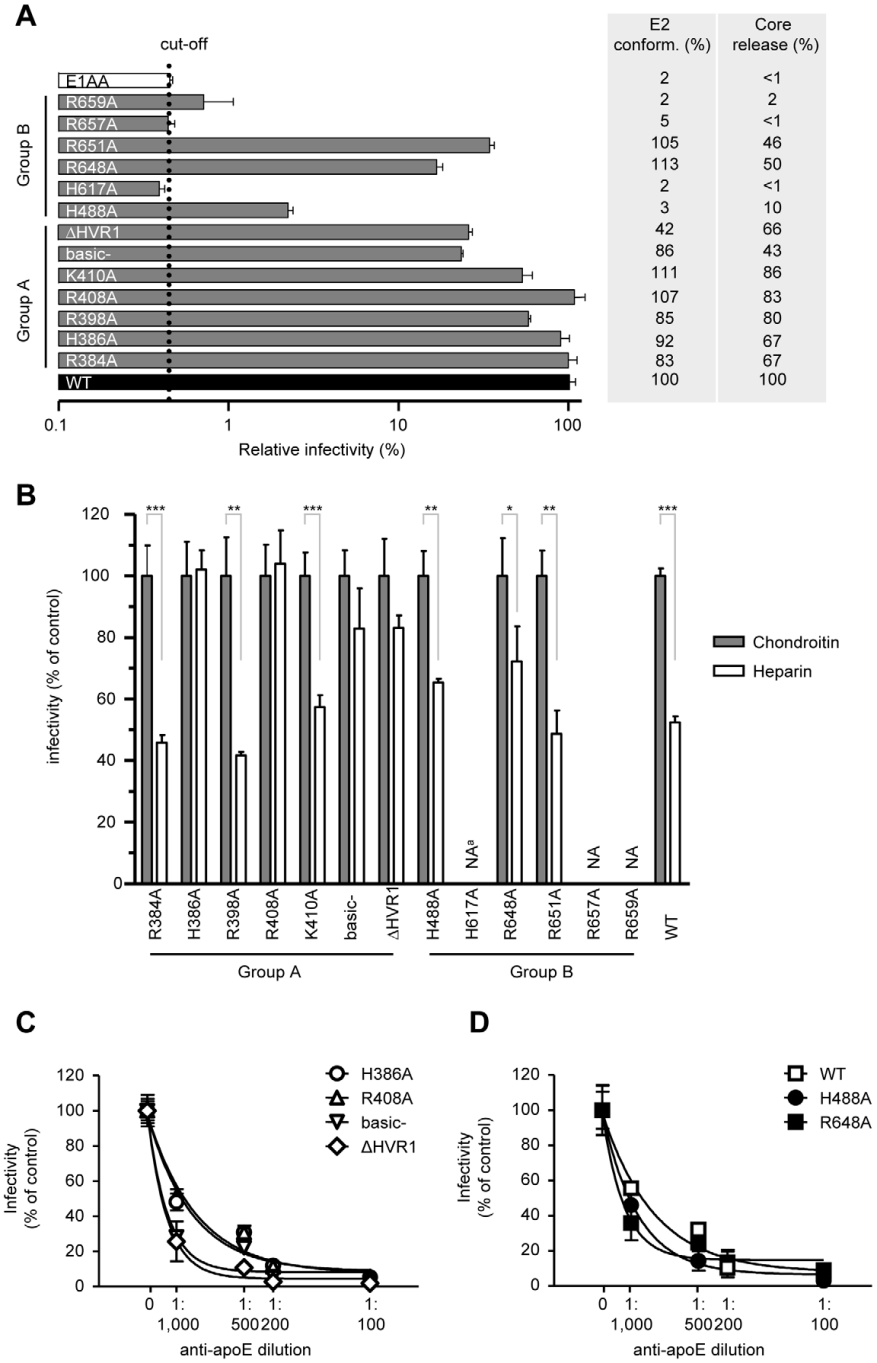
Effects of alanine substitutions for E2 basic amino acids on HCVcc infectivity, heparin neutralization, core release and anti-ApoE inhibition. (A) HCVcc infectivity of Luc-Jc1 WT or mutants. Infectivity of each mutant is expressed as a percentage of the infectivity level observed for the WT. The cut-off was set according to the infectivity observed for the E1AA mutant. Values shown represent the mean for three assays (± SD). Columns next to infectivity rates represent percentage (%) of E2 protein immunoprecipitated (shown in Fig. 2) and core supernatant release relative to WT virus. (B) Huh-7.5 cells were infected with Luc-Jc1 WT or with the indicated E2 mutant viruses in the presence of GAG antagonists (chondroitin sulfate or heparin, 200 µg/ml). The heparin neutralization scale was set relative to chondroitin sulfate neutralization. (C & D) Dose-dependent inhibition of mutantś entry by anti-ApoE. Infections were performed with WT or mutant viruses as follows: viruses were pre-incubated with anti-ApoE antibodies at the given concentrations and then added to the cells. Results are drawn from a representative experiment of three independent experiments. All points represent the mean of duplicate infections measured in duplicate (n = 4, ± SD).^a^ NA, not applicable.

Next, we tested whether alanine substitutions had any effect on HCV replication or infectivity. The mutations did not have any effect on replication (Fig. S1A). The results of our infectivity experiments are summarized in Figure 3A. Group A point mutants (R384A, H386A, R398A, K410A) either did not present any or had a moderate effect on infectivity while mutations of all basic amino acids (basic-) or deletion of HVR1 (ΔHVR1) lead to an infectivity reduction greater than 80%, in comparison to WT virus. For the group B mutants, only H488A, R648A and R651A mutants released infectious virus accompanied by a significant loss of infectivity. These results are in line with the core release in the supernatant of electroporated cells (Fig. 3A) and they also correlate well with the E2 conformation data (experimental data in Fig. 2B and quantification analysis in Fig. 3A) It is worth noting that mutants did not change the amount of secreted infectious virus relative to total infectious virus; although core protein secretion was affected by those mutants as deduced by core supernatant quantification (Fig. 3A). The amount of extracellular infectivity, relative to total infectivity, remained ∼90% (Fig. S1B) for those mutants that secreted viruses (Fig. 3A).

To determine the role of alanine mutants in E2-HS binding, we performed a series of Huh-7.5 cell infections, in the presence of chondroitin sulfate, which served as a non-antagonist to E2-HS interaction, or in the presence of heparin, which is known to be a strong antagonist [4]. Figure 3B summarizes our heparin inhibition results. H386A and R408A presented a total resistance to heparin inhibition while basic-, ΔHVR1, H488A and R648A mutations exhibited a less resistant phenotype to heparin. As the mutants E1AA, H617A, R657A and R659A did not produce infectious virus either extra- or intra-cellularly (Fig. S1B) infection experiments were not applicable (NA).

HCV particles are associated with the apolipoprotein E (ApoE) and possible other apolipoproteins [33]. Since ApoE can also bind to HS [34], we performed neutralization experiments using an anti-ApoE antibody and the previous identified heparin-resistant mutants (H386A, R408A, basic-, ΔHVR1, H488A and R648A). As shown in Figure 3C and 3D, a similar dose-dependent inhibition of infection was observed for WT and mutant viruses when HCVcc particles were pre-incubated with the anti-ApoE antibodies.

### Basic residues of E2 play a role in SR-BI binding

**Figure 4:**
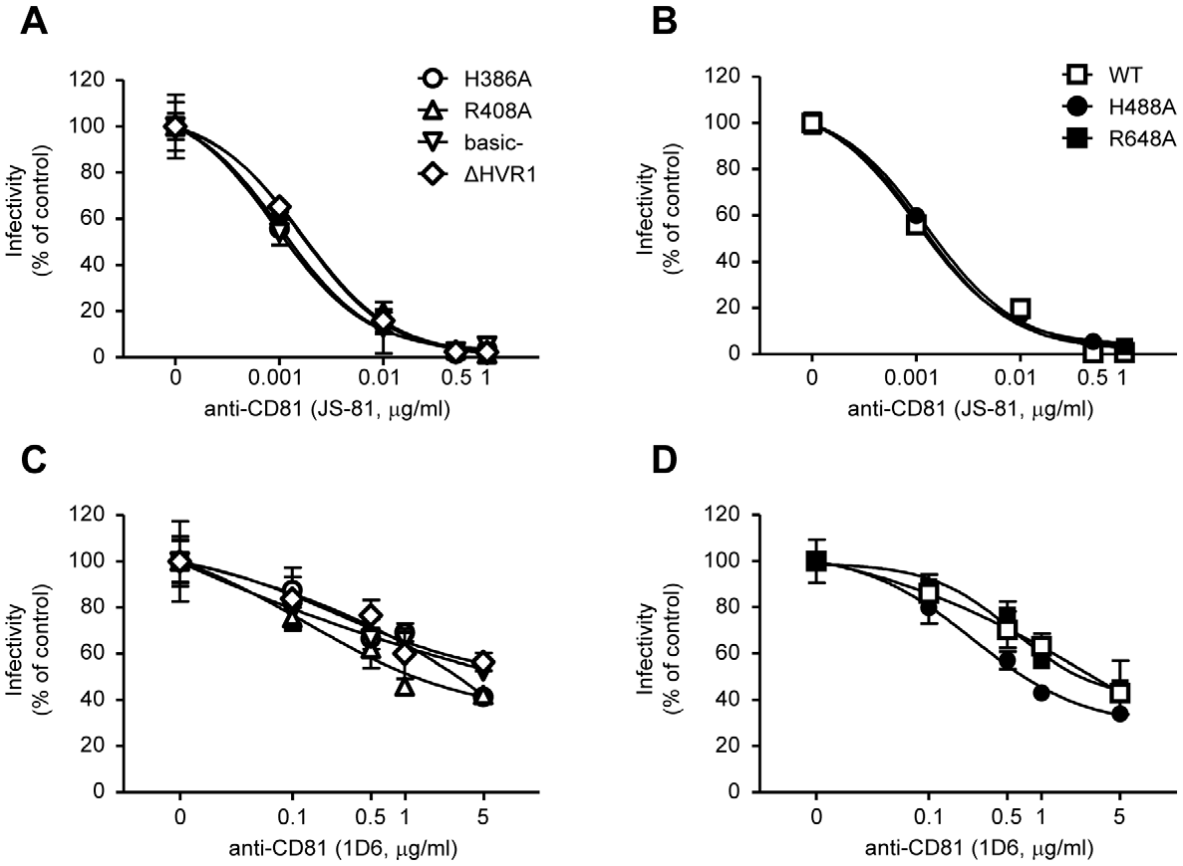
Dose-dependent inhibition of mutantś entry by anti-CD81 antibodies. Huh-7.5 cells were infected with Luc-Jc1 WT or with the indicated E2 mutant viruses as follows: viruses were added directly to the cells in the presence of anti-CD81 antibodies (clone JS81 or 1D6, A & B or C & D, respectively). Results are drawn from a representative experiment of three independent experiments. All points represent the mean of duplicate infections measured in duplicate (n = 4, ± SD).

To investigate the impact of the previously identified mutations on E2-CD81 interactions we exploited the ability of two anti-CD81 antibodies (clone JS-81 and clone 1D6) to inhibit WT or mutant infections. 1D6 inhibits E2-CD81 binding, whereas JS-81 may inhibit post-binding steps such as receptor oligomerization [35]. Figure 4A–D shows that all viruses presented a similar dose-dependent inhibition to both anti-CD81 antibodies.

**Figure 5:**
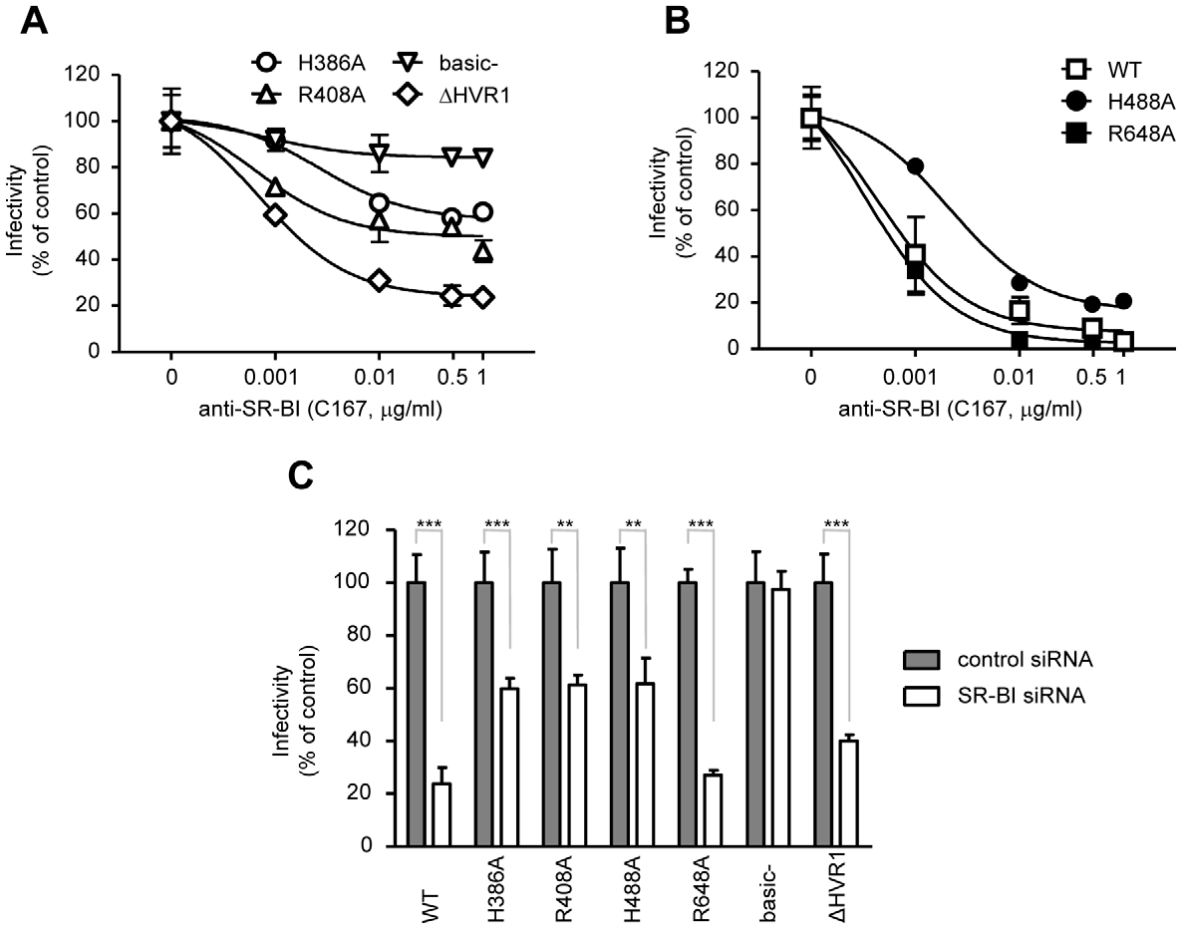
Dose-dependent inhibition of alanine mutants by anti-SR-BI and silencing of SR-BI. Huh-7.5 cells were infected with Luc-Jc1 WT or with the indicated E2 mutant viruses as follows: (A and B): in the presence of anti-SR-BI antibodies at the given concentrations. (C) Huh-7.5 cells were transfected 48 h prior to infection with control (scrambled) or SR-BI-specific siRNAs. Results are drawn from a representative experiment of three independent experiments. All points represent the mean of duplicate infections measured in duplicate (n = 4, ± SD).

Similar inhibition studies were done with two anti-SR-BI antibodies: the monoclonal C167 [36] and polyclonal IgG specific to SR-BI [37]. The basic- mutant presented a resistant phenotype to the anti-SR-BI antibody C167 (Fig. 5A), while the WT virus presented a dose-dependent inhibition (Fig. 5B). Notably, H386A and R408A mutants presented a partially resistant phenotype. Interestingly, the ΔHVR1 mutant remained sensitive to anti-SR-BI neutralization. Some degree of resistance was also observed for the H488A mutant, albeit at low anti-SR-BI concentrations. Similar results were obtained with the polyclonal anti-SR-BI IgG. However, the inhibition effect produced by those polyclonal antibodies was less potent than that obtained by the monoclonal C167 (Fig. S2).

To further analyze the role of SR-BI in WT and mutants entry, endogenous expression of SR-BI was silenced transiently with SR-BI-specific siRNAs prior to virus infection. SR-BI-specific siRNAs knocked down SR-BI endogenous expression by ∼60% (Fig. S3). Strikingly, the si-RNA-mediated knockdown of SR-BI did not affect basic- mutant entry while WT entry was inhibited more than 70% (Fig. 5C). Significantly, ΔHVR1 entry was also inhibited by SR-BI knock-down. Finally, we found partial entry inhibition for the mutants H386A, R408A and H488A which is in line with the neutralization studies with the anti-SR-BI antibodies.

### Basic residues of E2 protect HCV against neutralization by anti-E2 monoclonal and patient- derived IgG

**Figure 6:**
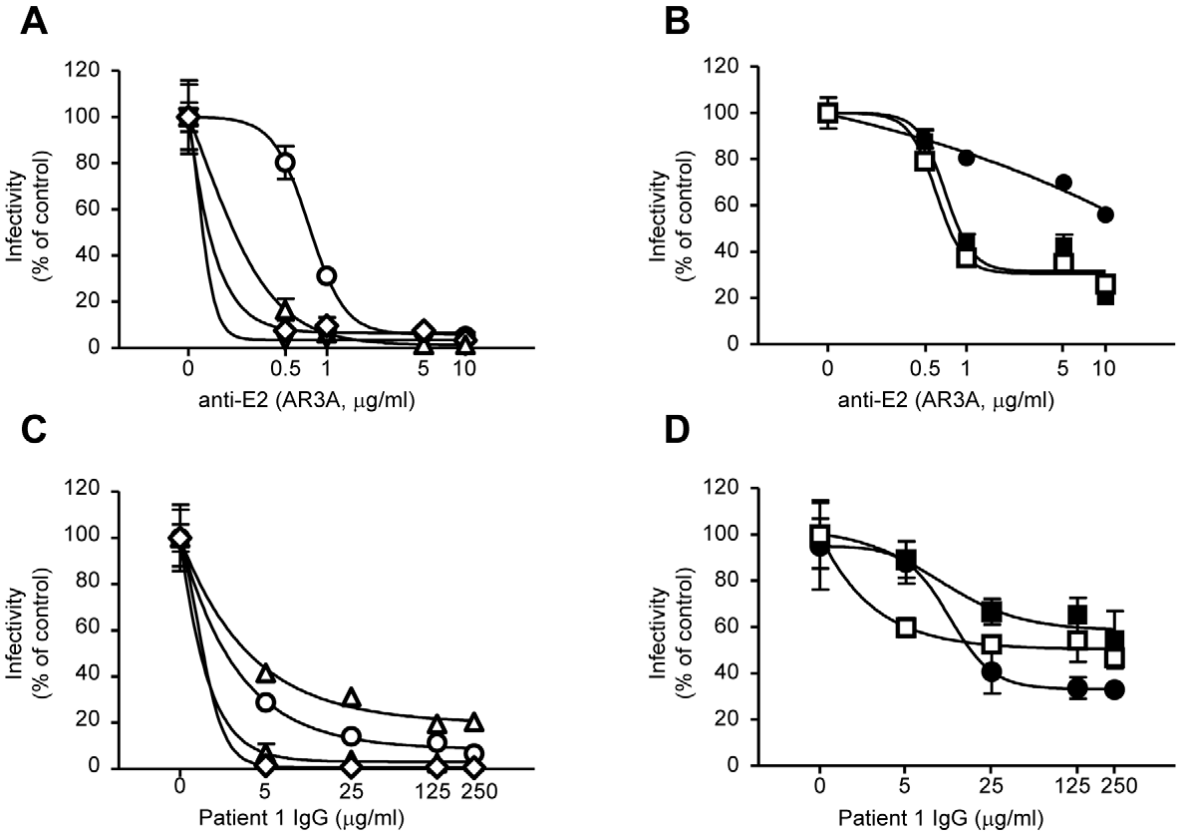
Dose-dependent inhibition of alanine mutants by conformational anti-E2 AR3A antibodies and patient-derived IgG. Huh-7.5 cells were infected with Luc-Jc1 WT or with the indicated E2 mutant viruses as follows: viruses were with pre-incubated with anti-E2 AR3A antibodies (A & B) or patient-derived IgG (C & D), at the given concentrations. Results are drawn from a representative experiment of three independent experiments. All points represent the mean of duplicate infections measured in duplicate (n = 4, ± SD).

To examine the neutralizing potential of the conformational anti-E2 AR3A antibody [32], we performed infections using pre-incubated viruses with various AR3A doses (Fig. 6A and 6B). This anti-E2 antibody exhibited ∼70% neutralization to the WT viruses at 5 µg/ml. Lower doses presented a ∼10–20% neutralization. However, basic-, H386A, and R408A mutants exhibited more than ∼95% neutralization at the higher doses, whereas basic- and R408A mutants presented a similar phenotype at the lower doses as well. Lastly, ΔHVR1 also presented increased sensitivity to anti-E2 neutralization. In contrast, the H488A mutant presented some resistance to this anti-E2 antibody. Similar results were obtained when viruses were pre-incubated with different amounts of polyclonal IgG derived by 2 chronic genotype 1b HCV patients [38] (Fig. 6C and 6D for patient 1, data not shown for patient 2), suggesting that basic amino acids protect conserved viral cross-neutralizing epitopes.

### Alanine mutants of basic residues of E2 affect HCVcc buoyant density

**Figure 7:**
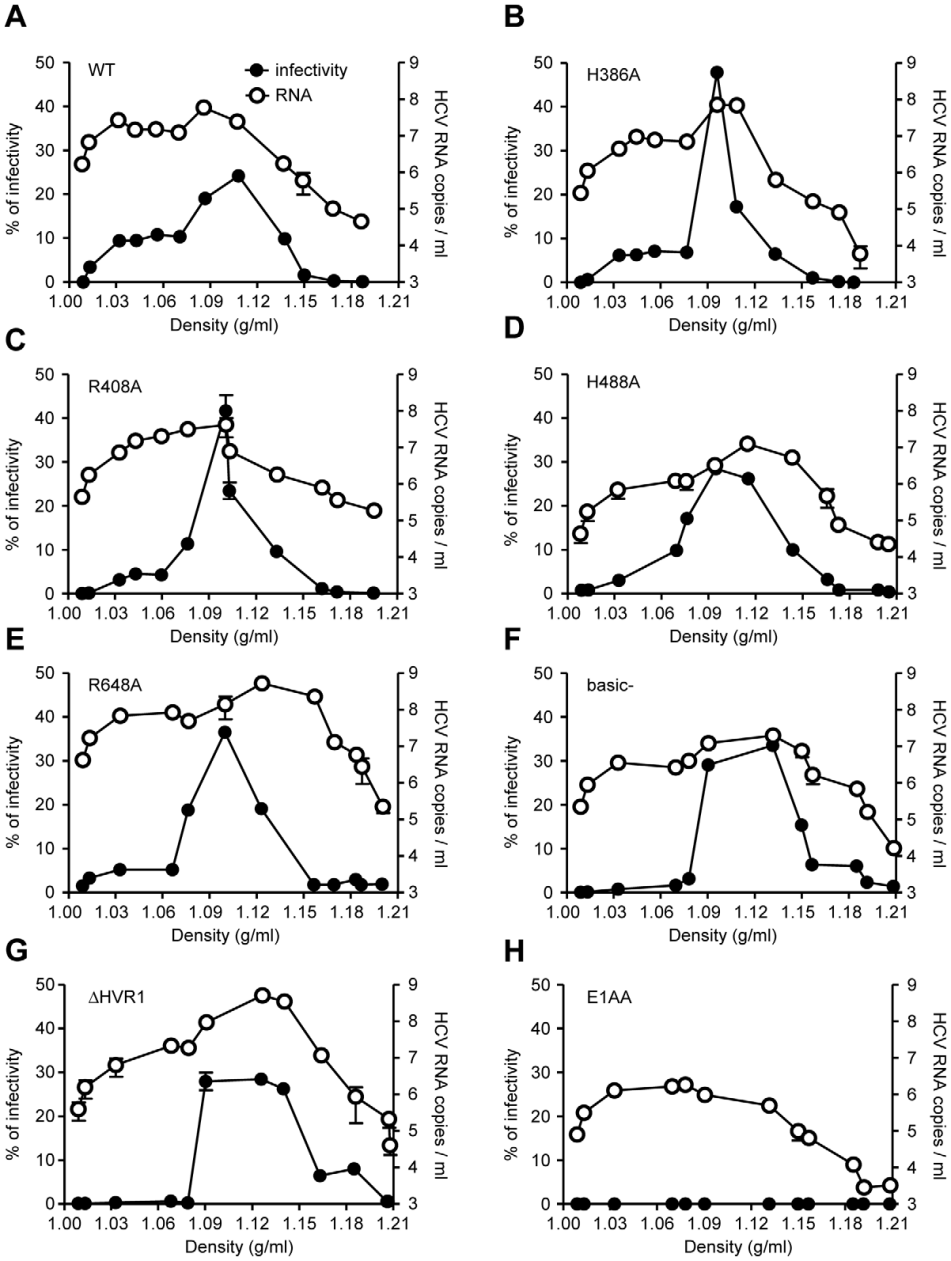
Buoyant density infectivity and RNA of WT and mutant viruses. The indicated viruses were resolved using an iodixanol step gradient. For each fraction HCV infectivity in Huh-7.5 cells and RNA (RT-qPCR) were determined. Values are plotted against the density of the respective fraction. The infectivity is expressed as a percentage of the total infectivity obtained from all fractions. All points represent the mean of duplicate infections measured in duplicate (n = 4, ± SD). Results are drawn from a representative experiment of two independent experiments.

To assess whether alanine mutants modulate the relationship between HCV and lipids or lipoproteins, we compared the buoyant density between WT and alanine mutants. Similar to previous studies [22], WT viruses presented a broad infectivity spectrum, ranging from 1.02 to 1.18 g/ml (Fig. 7A). The low-density viruses represented the HCV-lipoprotein complexes, and the infectivity peak occurred at ∼1.10 g/ml. HCV RNA (Fig. 7A) and core analysis (Fig. S4A) of the distinct HCV populations also confirmed previous results [22].

H386A, R408A, H488A, and R648A presented partial infectivity losses of the low-density viruses, resulting in sharper peaks of 1.10 g/ml (Fig. 7B–E). However, basic- and ΔHVR1 mutants exhibited a strong impairment in infectivity at lower densities. Thus, most of the detectable infectivity of these mutants reflected the virus particle types of intermediate density (Fig. 7F and 7G, respectively). HCV RNA analysis of mutant viruses revealed a shift of RNA-containing particles to higher densities for all mutants (Fig. 7B–G) Interestingly, Huh-7.5 cells transfected with the E1AA mutant, secreted non-infectious HCV-RNA containing structures (Fig. 7H) albeit at low levels, representing a ∼0.1–1% of total HCV-RNA secreted particles when compared to WT.

### HCV-associated lipoproteins protect viral cross-neutralizing epitopes and promote SR-BI-dependent entry

**Figure 8:**
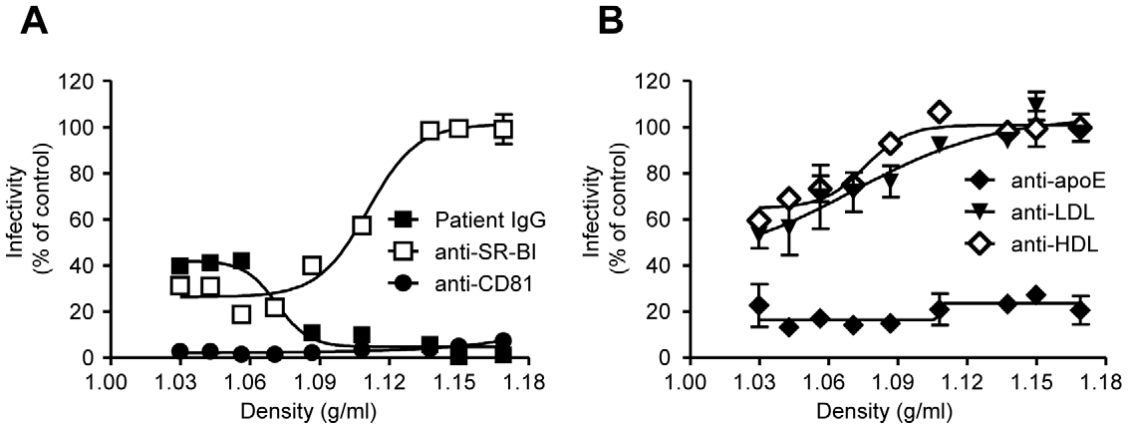
Density fraction neutralization of WT viruses by patient IgG, anti-SR-BI, anti-CD81, anti-LDL and anti-HDL. WT viruses were resolved using an iodixanol step gradient. (A) Infections of individual fractions in the presence of patient IgG, anti-SR-BI (C167) or anti-CD81 (JS-81). (B) Similar infections as described in (A) were performed with pre-incubated viruses with anti-ApoE, anti-HDL or anti-LDL. All points represent the mean of duplicate infections measured in duplicate (n = 4, ± SD). Results are drawn from a representative experiment of two independent experiments.

Initially, the specificity of commercial anti-LDL, anti-HDL and anti-ApoE antibodies with respect to their neutralization potential of WT Luc-Jc1 viruses was analyzed (Fig. S4). The half maximal inhibitory concentration (IC_50_) was ∼0.54 µg/ml and ∼8.09 µg/ml for the anti-LDL and anti-HDL antibodies respectively, while for the anti-ApoE the half maximal inhibitory dilution (ID_50_) was ∼1∶1,400. To clarify the mechanism underlying WT virus protection from patient IgG, density fractions of WT viruses were neutralized with IgG prior to infection. As shown in Figure 8A, low-density particles were partially protected, while medium or high density particles were potently neutralized, suggesting that lipoproteins protected conserved viral cross-neutralizing epitopes. Conversely, low-density particles were much more prone to anti-SR-BI, anti-HDL and anti-LDL neutralization (Fig. 8B), indicating that HCV-associated lipoproteins, rather than the virus itself, interact with this viral receptor. Surprisingly, anti-ApoE antibodies neutralized all WT fractions to the same extent, similar to anti-CD81 neutralizations (Fig. 8A and 8B).These results suggest that CD81 receptor usage is indispensable by all fractions and regardless the ApoE-associated amount to HCV, inhibition by anti-ApoE antibodies is highly robust. Our efforts to analyze the ApoE-associated amounts with respect to HCV density fractions was not successful because of the adequate expression and secretion of ApoE due to the cell metabolism: naïve Huh-7.5 cells secret similar ApoE amounts to Huh-7.5 cells as deduced by ApoE western blot analysis in density fractions. (Fig. S5B)

## Discussion

HS or other GAGs are used as receptors in the entry process of various members of the *Flaviviridae* family: dengue fever [39], classical swine fever (CSF) [40], and tick-borne encephalitis (TBE) [41]. Virus-GAGs interactions are mainly electrostatic and occur when the basic residues of the virus glycoproteins interact with negatively charged sulfated chains of the GAGs. In order to examine the role of the envelope glycoprotein E2 conserved basic amino acids, we generated a panel of alanine mutants for the *in vitro* infectious chimeric virus Jc1 (Table 1). These changes did not have any effect on virus replication. Initially, we analyzed E2 mutant conformation by immunoprecipitation and western blot with E2-specific antibodies. Three mutants (H617A, R657A and R659A) were not immunoprecipitated by this method suggesting that significant conformational changes are associated with the alanine mutations. These mutants did not also secrete infectious viruses and therefore were not analyzed further. For all other mutants (except the H488A), E2 expression and conformation remained at levels comparable to the WT virus. Interestingly, the basic- mutant, which harbours the maximum change in positive charge, did not demonstrate conformational change. Although we tried hard to analyze E1E2 heterodimerization, we failed to identify a suitable anti-E1 antibody for the Jc1 isolate. Further addressing of E2 conformation would have been interesting but outside the scope of this article.

HCVcc of the JFH-1 isolate (genotype 2a) interacts with heparin and it can be precipitated in a heparin-pulldown assay [42]. Although we did not provide direct evidence for HCV-heparin interactions, we identified two predominant positively-charged residues (H^386^ and R^408^) of the HVR1 and the highly conserved H^488^ and R^648^ amino acids in the putative GAG-binding motifs described by Olenina *et. al*, which are implicated in a heparin-resistance phenotype. Furthermore, our anti-ApoE neutralizations suggest that all mutants still carry ApoE and imply that the heparin-resistance phenotype is rather due to loss of direct E2-heparin and not to HCV-ApoE-heparin interactions.

Using serum-derived HCV, it has been suggested that the virus associated ApoB-containing lipoproteins rather than the E2 interact with SR-BI [17]. In our analyses, we used the anti-SR-BI monoclonal antibody C167, which not only prevents HDL binding to SR-BI, but also inhibits SR-BI-mediated cholesterol efflux. As shown in Figure 8A, our data strongly support this hypothesis: viral particles of low density were neutralized by anti-SR-BI, anti-LDL and anti-HDL antibodies. Furthermore, we demonstrate that a mutant virus lacking basic residues in the HVR1 was able to gain entry in an anti-SR-BI-independent fashion. This mechanism was additive and dependent upon the positive charge of HVR1. Strikingly, we inhibited ΔHVR1 mutant entry, albeit up to only ∼80%, while inhibition of WT was almost complete. Bankwitz *et al.* [22], were not able to inhibit ΔHVR1 viruses with polyclonal anti-SR-BI IgG. The lower inhibition potency of these polyclonal antibodies compared to the monoclonal C167 antibodies (Fig. S2), may explain this discrepancy. Our SR-BI knockdown studies were totally in line with the anti-SR-BI neutralization studies. These findings suggest an exclusion of SR-BI receptor for the basic- mutant entry. HVR1 has been also implicated in the SR-BI-mediated entry [22]. However, our results concerning ΔHVR1 neutralizations and entry inhibitions by SR-BI knockdown suggest that E2-SR-BI interaction is complex and cannot be restricted to HVR1.

The confirmed epitope of the A3RA antibody is formed by the three segments spanning amino acids 396–424, 436–447, and 523–540 [32]; the first segment also overlaps with the HVR1 region (aa 384–410). Mutants R408A, basic- and ΔHVR1 were effectively neutralized at low doses and H386A presented a similar phenotype at higher doses, suggesting that the absence of HVR1 (or its positive charge) led to the antigenic exposure of the other two segments. Consistent with these observations, our results also underscore the important properties of basic residues about protection against neutralizing antibodies. Cross-neutralization of WT HCVcc by patient-derived IgG was limited, but much more efficient when the basic amino acids of HVR1 were mutated or when the HVR1 was absent.

HCV particles carrying the H488A or the R648A mutations, or lacking basic amino acids in the HVR1 or the HVR1 itself, differed from WT in the following regards: the very low-density range WT particles were infectious; however, infectivity capacities of H386A, R408A, H488A and R648A mutants were noted primarily in the intermediate densities and for mutants basic- and ΔHVR1 only in the intermediate and high densities. *In vivo*, the variable densities of HCV particles have been attributed to differential associations with lipoproteins and antibodies [19]. HCVcc also associates with lipoproteins, thus exhibiting a heterogeneous density [11], [33]. Our data suggest that the positive charge of HVR1, as well as those charges of the highly conserved H^488^ and R^648^, is involved in HCV-lipoprotein interactions. Moreover, the resistance of the basic- mutant to anti-SR-BI inhibition further supports our hypothesis that virus-associated lipoproteins, rather than E2, interact with SR-BI. Furthermore, our observations are consistent with a previous study demonstrating that HDL is a serum factor that attenuates neutralization of HCVpp or HCVcc [21] by antiviral antibodies in a SR-BI dependent manner. Here we demonstrate that mutants resistant to anti-SR-BI inhibition were at the same time more sensitive to anti-E2 neutralization. Based on our results with regard to WT viruses shown in Figure 8B, we strongly contend that the viral-associated lipoproteins are the key partner for all of these interactions and their subsequent effects. These findings indicate that there exists some interplay between SR-BI-HCV (and its lipoproteins) and neutralizing antibodies

Finally, our data could provide valuable information for the design and development of HCV entry inhibitors. Studies in uPA-SCID mice have shown that HCV infection can be prevented *in vivo* by blocking the CD81 or the SR-BI receptors with the monoclonal antibodies JS-81 or novel SR-BI-specific antibodies, respectively [43], [44]. Although a HCV CD81-escape variant has not been ever reported, CD81 is not involved in direct cell-to-cell transmission. Furthermore, as we report here SR-BI-independent variants, our results are critical for the design of any HCV preventing strategy based on SR-BI [44]. A more favourable approach could be therefore a combination therapy, targeting both HCV receptor(s) and viral envelope protein(s).

## Supporting Information

Information S1 **A detailed list of antibodies used in this study and protocols for site-directed mutagenesis, flow cytometry and HCV cell culture replication kinetics determination are provided in this section.**
(DOC)

Figure S1 **Effect of E2 mutants on HCV replication and virus particle production.** (A) Huh-7.5 cells were electroporated with RNAs of Luc-Jc1 variants specified at the bottom. Luciferase activity was measured at the indicated time points and is expressed relative to RLUs obtained 4 h post transfection. Each bar present the mean of duplicate wells measured in duplicate with standard deviation (n = 4, ± SD). (B) Huh-7.5 cells were electroporated with RNAs of Jc1 variants specified at bottom. 72 h post electroporation, virus titers were measured by TCID_50_ in the supernatant (extracellular infectivity) or within the cells after 3 rounds of freeze-and-thaw (intracellular infectivity). Results are drawn from a representative experiment of three independent experiments and are expressed as a percentage of total infectivity (intra- and extracellular) with standard deviations.
(TIF)

Figure S2 **Inhibition of mutantś entry by anti-SR-BI antibodies.** Huh-7.5 cells were infected with WT, “basic-”, or ΔHVR1 viruses in the presence of anti-SR-BI antibodies (human monoclonal C167 or rat polyclonal anti-SR-BI). Results are expressed relative to control inhibitions with human or rat IgGs for the C167 or the rat anti-SR-BI, respectively. Results are drawn from a representative experiment of three independent experiments. All points represent the mean of duplicate infections measured in duplicate with standard deviations (n = 4, ± SD).
(TIF)

Figure S3 **Expression of SR-BI on the surface of Huh-7.5 cells.** SR-BI expression was analyzed 48 h post-transfection with control or SR-BI-specific siRNAs. Cells were stained by using SR-BI-specific (C167) antibodies and secondary antibodies conjugated with Alexa Fluor® 488. Gray profiles represent cells that were stained only with the secondary antibodies. MFI: Mean fluorescence intensity.
(TIF)

Figure S4 **Specificity evaluation of anti-LDL, anti-HDL and anti-ApoE against WT Luc-Jc1 viruses.** Luc-Jc1 viruses were pre-incubated 1 h at RT with anti-LDL, anti-HDL (A) or anti-ApoE antibodies (B), at the given concentrations or dilutions, respectively. Infections with pre-incubated viruses were performed as described in the main text. Infectivity of each condition is expressed as a percentage of the infectivity level observed for the control antibodies. Results are drawn from a representative experiment of three independent experiments. All points represent the mean of duplicate infections measured in duplicate (n = 4, ± SD).
(TIF)

Figure S5 **ApoE and core detection in density fractions.** Huh-7.5 cells were electroporated with WT or with the indicated E2 mutant viruses. 72 h post electroporation supernatants were harvested and separated in an iodixanol density gradient. Fractions of WT viruses were plotted for ApoE and core proteins (A) while mutant viruses were plotted only for ApoE. Supernatant from mock electroporated cells served for detection of the ApoE constitutive cell secretion.
(TIF)
