# Supplementary material for: Interplay between Basic Residues of Hepatitis C Virus Glycoprotein E2 with Viral Receptors, Neutralizing Antibodies and Lipoproteins
Source: PLoS One. 2012 Dec 27;7(12):e52651. doi: 10.1371/journal.pone.0052651 (PMC3531341; doi:10.1371/journal.pone.0052651)
Supplement: Information S1 — A detailed list of antibodies used in this study and protocols for site-directed mutagenesis, flow cytometry and HCV cell culture replication kinetics determination are provided in this section. (DOC) [file pone.0052651.s001.doc]

# Supporting Information S1

# Supporting Materials and Methods

*Antibodies*

- anti-E2 human conformational mAb AR3A (kindly provided by Dr. M. Law)
- anti-E2 mouse mAb BDI167 (Santa Cruz Biotechnology, Santa Cruz, CA)
- anti-CD81 mouse mAb clone JS-81 (BD Pharmingen, San Diego, CA)
- anti-CD81 mouse mAb 1D6 (**Fitzgerald Industries International**, North
  Acton, MA)
- anti-SR-BI (clone C167, kindly provided by Dr. A. Nicosia)
- anti-SR-BI and negative control polyclonal rat antibodies (kindly provided by Dr. T. Baumert)
- anti-β-actin mouse mAb (clone 6C5, Applied Biosystems, Foster city, CA)
- HRP-conjugated goat anti-mouse mAb (Fc specific, Sigma-Aldrich, St. Louis, MO)
- mouse IgG_1_, κ isotype control clone MOPC-31C, (BD Pharmingen, San Diego, CA)
- anti-apoE goat pAb for neutralizations (Calbiochem-Merck KGaA, Darmstadt, Germany)
- anti-apoE goat pAb for WB (Abnova, GmbH, Heidelberg, Germany)
- goat normal serum (GNS) (Jackson Immunoresearch, Suffold, UK)
- anti-LDL mouse mAb (clone M-34, Progen, Heidelberg, Germany)
- anti-HDL mouse mAb (clone M-32, Progen, Heidelberg, Germany).

*Site-directed mutagenesis*

Alanine mutants were introduced into the HC-J6CH E1E2 expression plasmid (pcDNA3.1-ΔcE1E2-J6CH using the PCR-based GENEART® Site-Directed Mutagenesis System (Invitrogen Eugene, OR) with a slight protocol modification: KOD Hot Start DNA Polymerase (Merck KGaA, Darmstadt, Germany) was utilized instead of the recommended polymerases. Primers designed according to the manufacturer’s protocol are summarized in Suppl. Table 2. Mutations were confirmed by BigDye 3.1 Terminator chemistry sequencing (Applied Biosystems, Foster City, CA, USA). Mutations introduced into the pcDNA3.1-E1E2-J6CH plasmid were transferred to HCVcc system plasmids (pFK-Jc1 and pFK-Luc-Jc1 , kindly provided by Prof. R. Bartenschlager) by ligating a *BsiW*I/*Aar*I 1824bp fragment into the pFK-Jc1 vector, previously excised from the pcDNA3.1-ΔcE1E2-J6CH vector. Subsequently, an *Nsi*I 4252bp fragment was excised from the pFK-Jc1-mutants and ligated to a previously linearized pFK-Luc-Jc1 plasmid with the same enzyme. The pFK-Luc-Jc1-ΔHVR1 plasmid was a gift from Dr. T. Pietschmann and the pFK-JFH1-SGR-Luc from Dr. R. Bartenschlager.

## HCV cell culture replication kinetics determination

Huh-7.5 cells were electroporated with WT or mutant Luc-Jc1 RNAs and diluted in 10 ml DMEM complete. 250 μl of cells from each virus were seeded in 12-well plates in duplicate. Cells were lysed for the indicated time points (Fig. S2) and luciferase activity was then measured.

*Flow cytometry*

Huh-7.5 cells were detached by using PBS supplemented with 0.2% (W/V) EDTA, washed twice with PBS, and passed through a 16-gauge needle. Approximately 5 × 10^5^ cells per ml were stained for 1 h at 4°C with SR-BI-specific monoclonal antibody (C167) diluted to 1 μg/ml in PBS containing 2% bovine serum albumin and 0.02% sodium azide (FACS sample buffer). Subsequently, cells were washed with PBS and bound antibodies were detected by incubation for 1 h at 4°C with human-specific secondary antibodies conjugated with Alexa Fluor® 488 (Invitrogen, Carlsbad, CA) at a dilution of 1:200 in FACS sample buffer. Stained cells were washed with PBS, resuspended in 500 μl FACS sample buffer, and analyzed immediately using a FACSCalibur apparatus and the Cell Quest Pro software (both from Becton Dickinson Biosciences).

## References

1. Schaller T, Appel N, Koutsoudakis G, Kallis S, Lohmann V, et al. (2007) Analysis of hepatitis C virus superinfection exclusion by using novel fluorochrome gene-tagged viral genomes. J Virol 81: 4591-4603.

2. Koutsoudakis G, Kaul A, Steinmann E, Kallis S, Lohmann V, et al. (2006) Characterization of the early steps of hepatitis C virus infection by using luciferase reporter viruses. J Virol 80: 5308-5320.
