# Supplementary figures and images for: Interplay between Basic Residues of Hepatitis C Virus Glycoprotein E2 with Viral Receptors, Neutralizing Antibodies and Lipoproteins
Source: PLoS One. 2012 Dec 27;7(12):e52651. doi: 10.1371/journal.pone.0052651 (PMC3531341; doi:10.1371/journal.pone.0052651)

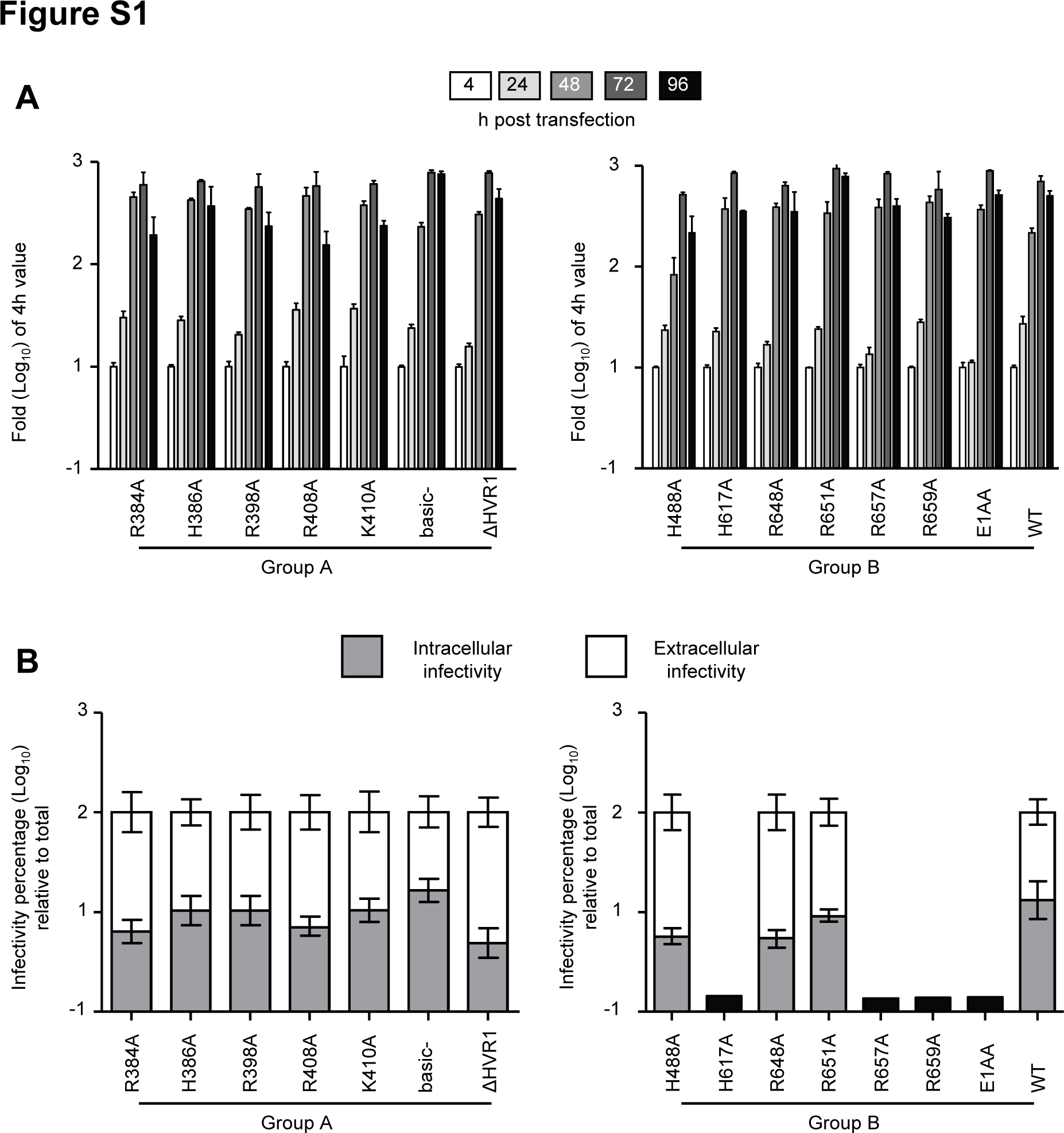

Supplement: Figure S1 — Effect of E2 mutants on HCV replication and virus particle production. (A) Huh-7.5 cells were electroporated with RNAs of Luc-Jc1 variants specified at the bottom. Luciferase activity was measured at the indicated time points and is expressed relative to RLUs obtained 4 h post transfection. Each bar present the mean of duplicate wells measured in duplicate with standard deviation (n = 4, ± SD). (B) Huh-7.5 cells were electroporated with RNAs of Jc1 variants specified at bottom. 72 h post electroporation, virus titers were measured by TCID50 in the supernatant (extracellular infectivity) or within the cells after 3 rounds of freeze-and-thaw (intracellular infectivity). Results are drawn from a representative experiment of three independent experiments and are expressed as a percentage of total infectivity (intra- and extracellular) with standard deviations. (TIF) [file pone.0052651.s002.tif]

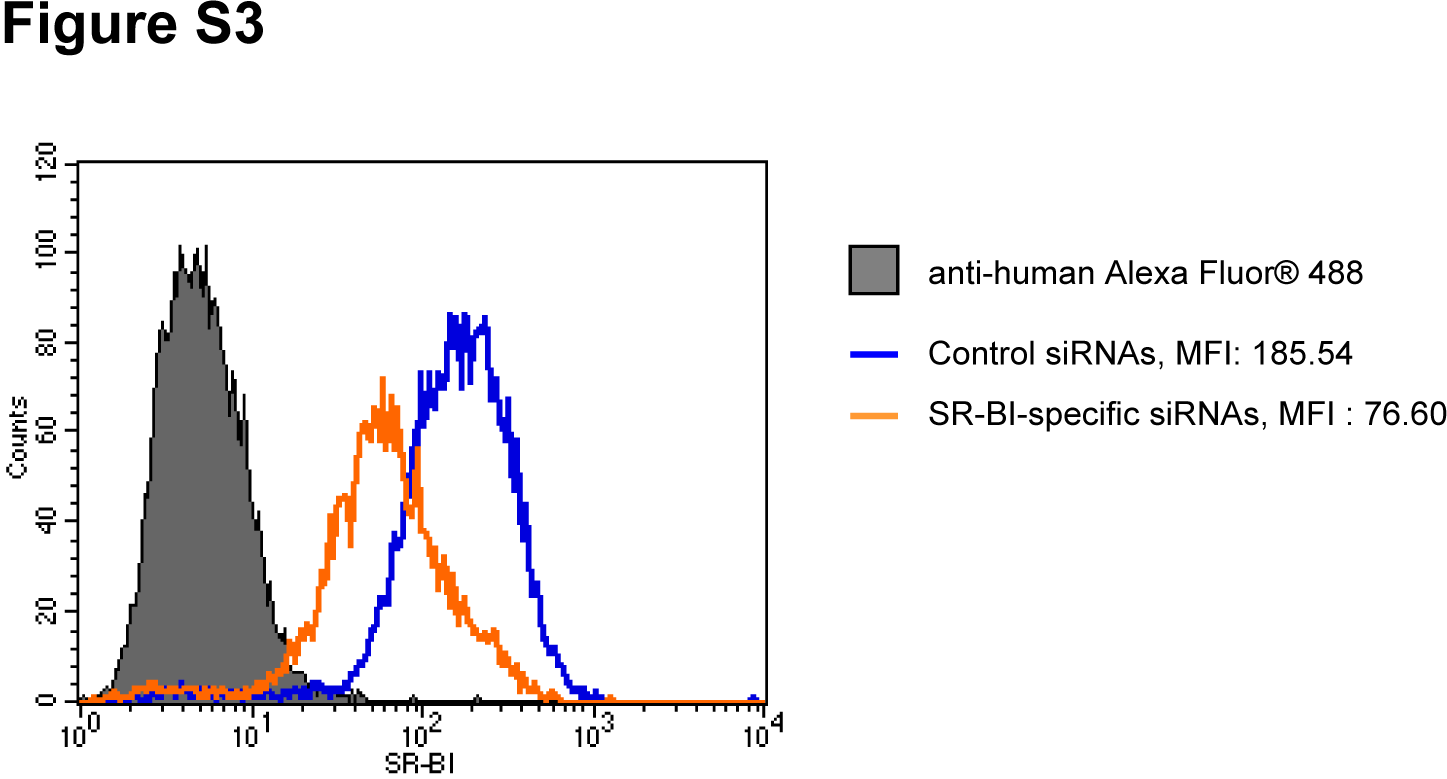

Supplement: Figure S2 — Inhibition of mutantś entry by anti-SR-BI antibodies. Huh-7.5 cells were infected with WT, “basic-”, or ΔHVR1 viruses in the presence of anti-SR-BI antibodies (human monoclonal C167 or rat polyclonal anti-SR-BI). Results are expressed relative to control inhibitions with human or rat IgGs for the C167 or the rat anti-SR-BI, respectively. Results are drawn from a representative experiment of three independent experiments. All points represent the mean of duplicate infections measured in duplicate with standard deviations (n = 4, ± SD). (TIF) [file pone.0052651.s003.tif]

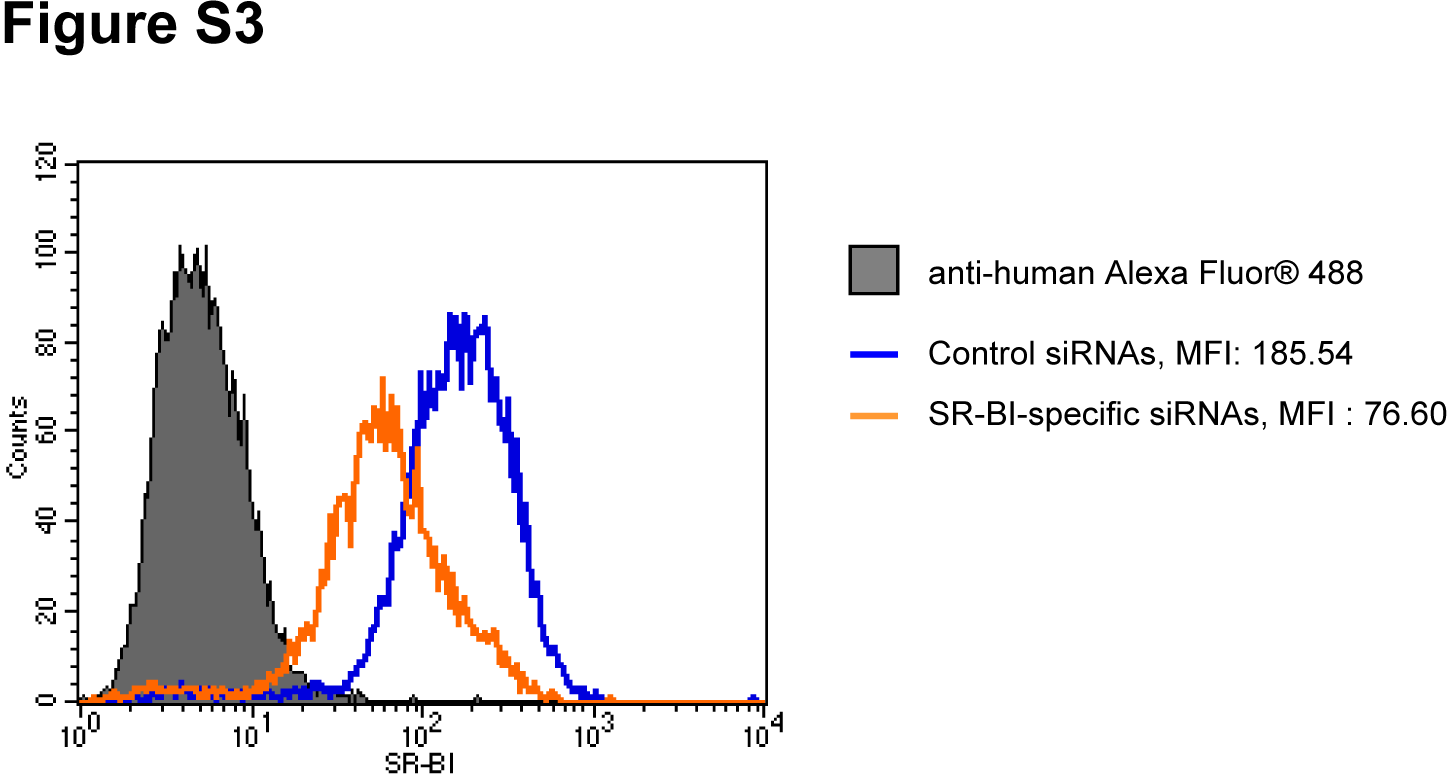

Supplement: Figure S3 — Expression of SR-BI on the surface of Huh-7.5 cells. SR-BI expression was analyzed 48 h post-transfection with control or SR-BI-specific siRNAs. Cells were stained by using SR-BI-specific (C167) antibodies and secondary antibodies conjugated with Alexa Fluor® 488. Gray profiles represent cells that were stained only with the secondary antibodies. MFI: Mean fluorescence intensity. (TIF) [file pone.0052651.s004.tif]

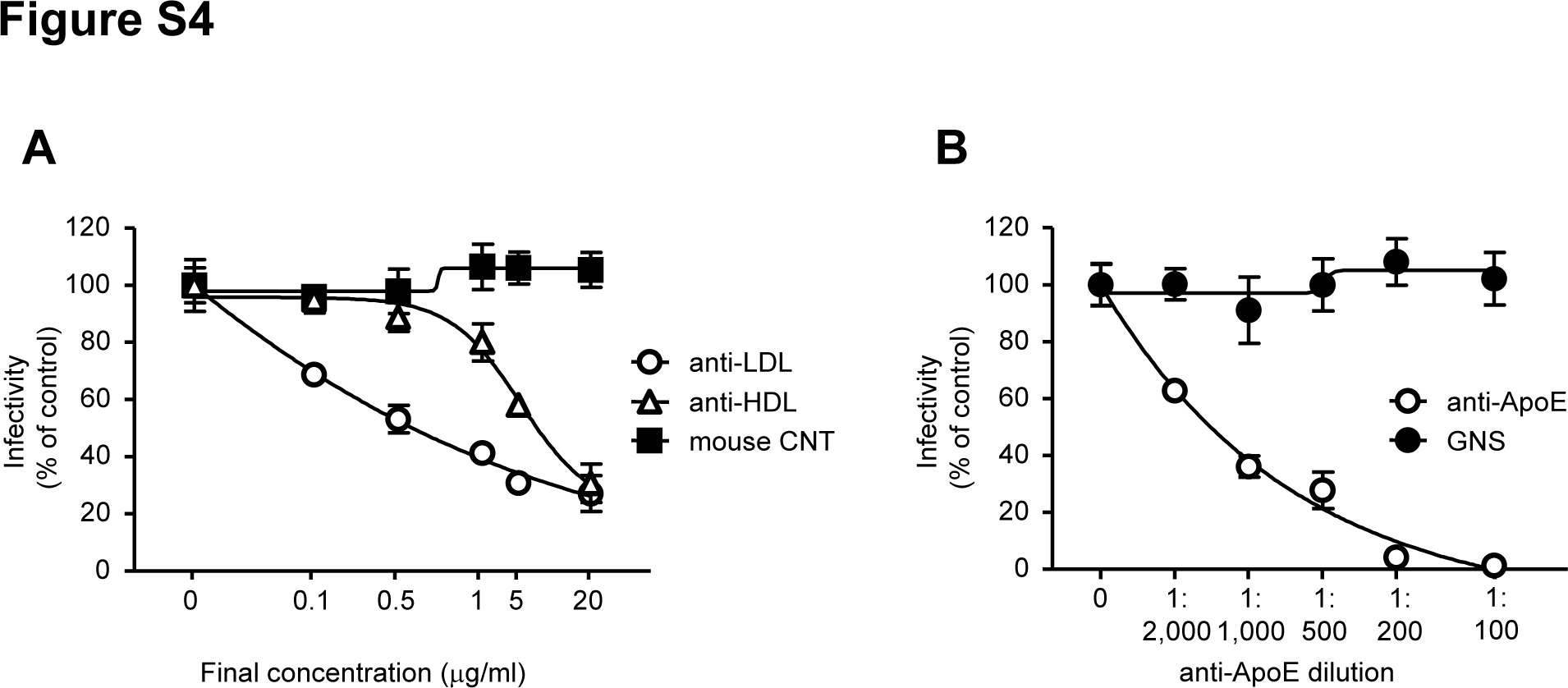

Supplement: Figure S4 — Specificity evaluation of anti-LDL, anti-HDL and anti-ApoE against WT Luc-Jc1 viruses. Luc-Jc1 viruses were pre-incubated 1 h at RT with anti-LDL, anti-HDL (A) or anti-ApoE antibodies (B), at the given concentrations or dilutions, respectively. Infections with pre-incubated viruses were performed as described in the main text. Infectivity of each condition is expressed as a percentage of the infectivity level observed for the control antibodies. Results are drawn from a representative experiment of three independent experiments. All points represent the mean of duplicate infections measured in duplicate (n = 4, ± SD). (TIF) [file pone.0052651.s005.tif]

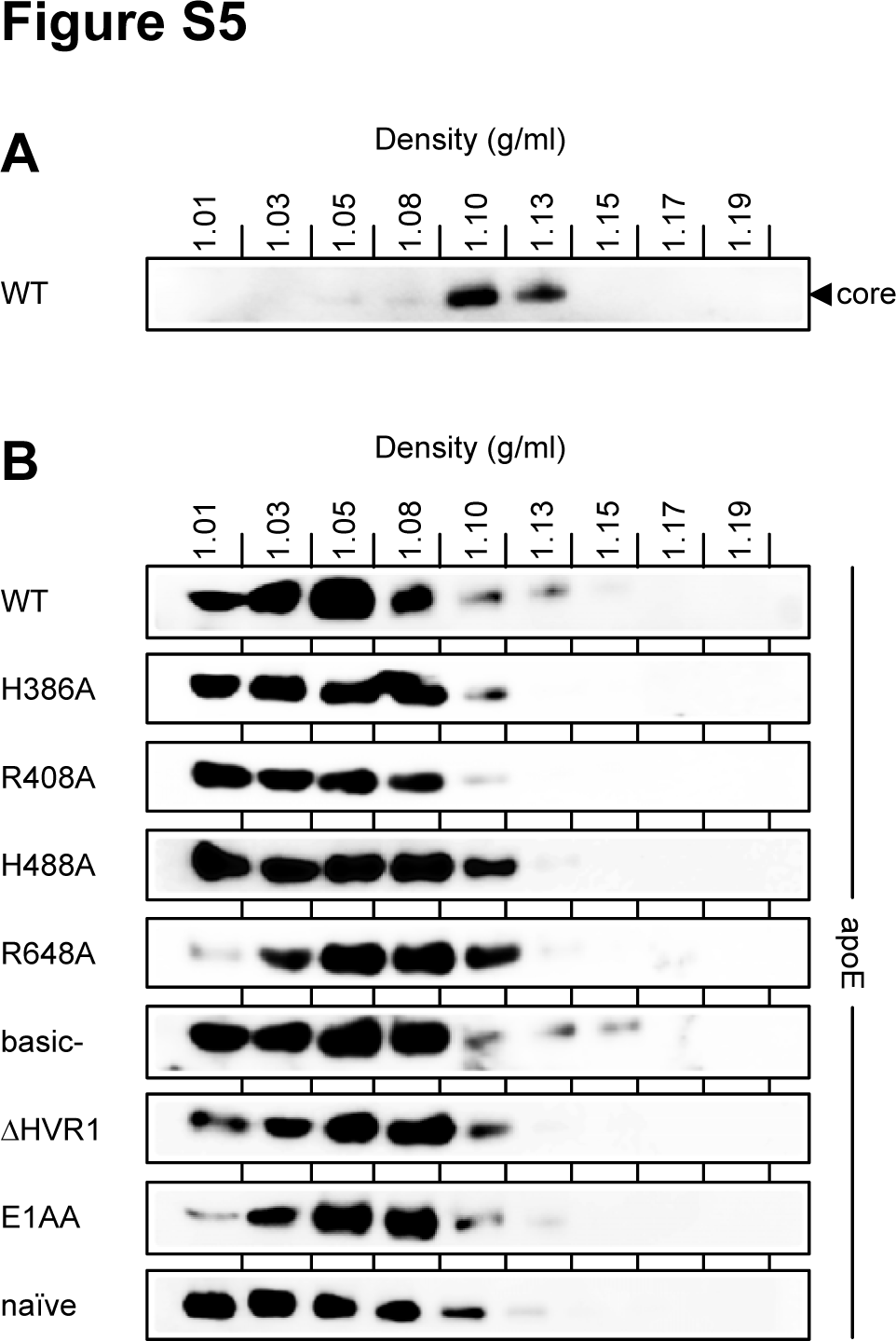

Supplement: Figure S5 — ApoE and core detection in density fractions. Huh-7.5 cells were electroporated with WT or with the indicated E2 mutant viruses. 72 h post electroporation supernatants were harvested and separated in an iodixanol density gradient. Fractions of WT viruses were plotted for ApoE and core proteins (A) while mutant viruses were plotted only for ApoE. Supernatant from mock electroporated cells served for detection of the ApoE constitutive cell secretion. (TIF) [file pone.0052651.s006.tif]
